# Chromosome-level genome provides insight into the evolution and conservation of the threatened goral (*Naemorhedus goral*)

**DOI:** 10.1186/s12864-024-09987-5

**Published:** 2024-01-22

**Authors:** Nan Sun, Xiao-Ying Ma, Guang-Hong Shi, Xiao-Hong Yang, Wei Li, Chen-Guang Feng, Da Mi, Guo-Gang Li, Ji-Qi Lu

**Affiliations:** 1 School of Life Sciences, Zhengzhou University, 450001 Zhengzhou, Henan, China; 2 College of Life Sciences, Academy of Plateau Science and Sustainability, Qinghai Normal University, 810008 Xining, Qinghai, China; 3 Qinghai Makehe Forestry Bureau, Golog Tibetan Autonomous Prefecture 814300, Qinghai, China; 4 Xi’an Haorui Genomics Technology Co., LTD, 710116 Xi’an, Shaanxi China; 5 School of Ecology and Environment, Northwestern Polytechnical University, 710129 Xi’an, Shaanxi China; 6 Key Laboratory of Biomedical Information Engineering of Ministry of Education, School of Life Science and Technology, Xi’an Jiaotong University, 710049 Xi’an, Shaanxi China

**Keywords:** Chromosome-level genome, *De novo* assembly, Goral, Phylogenetics, Conservation

## Abstract

**Background:**

Gorals *Naemorhedus* resemble both goats and antelopes, which prompts much debate about the intragenus species delimitation and phylogenetic status of the genus *Naemorhedus* within the subfamily Caprinae. Their evolution is believed to be linked to the uplift of the Qinghai-Tibet Plateau (QTP). To better understand its phylogenetics, the genetic information is worth being resolved.

**Results:**

Based on a sample from the eastern margin of QTP, we constructed the first reference genome for Himalayan goral *Naemorhedus goral*, using PacBio long-read sequencing and Hi-C technology. The 2.59 Gb assembled genome had a contig N50 of 3.70 Mb and scaffold N50 of 106.66 Mb, which anchored onto 28 pseudo chromosomes. A total of 20,145 protein-coding genes were predicted in the assembled genome, of which 99.93% were functionally annotated. Phylogenetically, the goral was closely related to muskox on the mitochondrial genome level and nested into the takin-muskox clade on the genome tree, rather than other so-called goat-antelopes. The cladogenetic event among muskox, takin and goral occurred sequentially during the late Miocene (~ 11 − 5 Mya), when the QTP experienced a third dramatic uplift with consequent profound changes in climate and environment. Several chromosome fusions and translocations were observed between goral and takin/muskox. The expanded gene families in the goral genome were mainly related to the metabolism of drugs and diseases, so as the positive selected genes. The *Ne* of goral continued to decrease since ~ 1 Mya during the Pleistocene with active glaciations.

**Conclusion:**

The high-quality goral genome provides insights into the evolution and valuable information for the conservation of this threatened group.

**Supplementary Information:**

The online version contains supplementary material available at 10.1186/s12864-024-09987-5.

## Introduction

Gorals *Naemorhedus* belongs to the subfamily of Caprinae (Bovidae), resembling both goat and antelopes. According to the International Union for Conservation of Nature (IUCN), goral species are assessed as ‘Vulnerable’ and ‘Near Threatened’ due to a declining population caused by poaching and destruction of forested mountain habitats [1–3]. The distribution and evolution of gorals are believed to be linked to the uplift of the Qinghai-Tibet Plateau (QTP) [4], where the most chaotic assessment of goral phylogenetics is found across the southeastern margin, at the junction of tremendous Himalayas and Hengduan Mountains. However, with the solitary and elusive habit, gorals are poorly known, not separated from serows (*Capricornis*) as an independent genus until three decades ago [5]. Even now, many locals still believe that gorals are the offspring of serows (private survey) due to their similar looking and sympatric distribution. It prompts much debate about the intragenus species delimitation and phylogenetic status of the genus *Naemorhedus* within the subfamily Caprinae.

Within the genus, seven goral species have been proposed, containing *N. goral*, *N. griseus*, *N. caudatus*, *N. baileyi*, *N. bedfordi*, *N. evansi* and *N. cranbrooki*, based on morphological and ecological characteristics. Having experienced splitting and lumping, the number of goral species varied from three to six [5–7]. Recently, in the context of the phylogenetic species concept, five goral species are recognized based on complete mitochondrial genomes [4] and six species using gene markers (i.e. Cyt *b*, D-loop) [8], due to differences in genetic data integrity. In the subfamily Caprinae, goral is considered morphologically belonging to the tribe Rupicaprini, so-called goat-antelopes, together with serow from south to east Asia, chamois *Rupicapra* from Europe to west Asia, and Rocky mountain goat *Oreamnos* from North America [9]. However, a similar topology at the mitochondrial genome level is recovered that goral and serow, as sister genera, are much closer to muskox *Ovibos moschatus* than chamois and mountain goat, while the later two clustered with takin *Budorcas taxicolor* in another clade [10–14]. Inconsistently, muskox and takin, formerly as the tribe Ovibovini, are clustered and far from mountain goats on the genome tree [15]. The status of goral is still uncertain due to data absence.

The bottlenecks encountered in current research suggest the limited explanatory power of the mitochondrial genome to reconstruct phylogenetic relationships, which is not only the foundation of taxonomy, but also the premise of revealing the mechanism of speciation and evolution [16]. To a certain extent, genome trees could be better to reflect the overall evolutionary process of species and be closer to species trees [17]. Thus, genomic-scale data is necessary. Benefiting from the rapid development of sequencing technology, dozens of reference genomes in subfamily Caprinae have been released in the GenBank database, among which only eight have been assembled at chromosome-level and none are available for goat-antelopes to date (https://www.ncbi.nlm.nih.gov/). It limits us to understand the internal phylogeny and evolution of this important group. In this study, the first genome of goral is assembled at the chromosome level using PacBio sequel II long reads and Hi-C technology. The preliminary bioinformatic analyses are also performed. It will serve as a high-quality reference genome, provide valuable information for further genetic analysis and benefit the evolutionary and conservation studies for goral species.

## Results

### Chromosome-level genome assembly and annotation

**Fig. 1:**
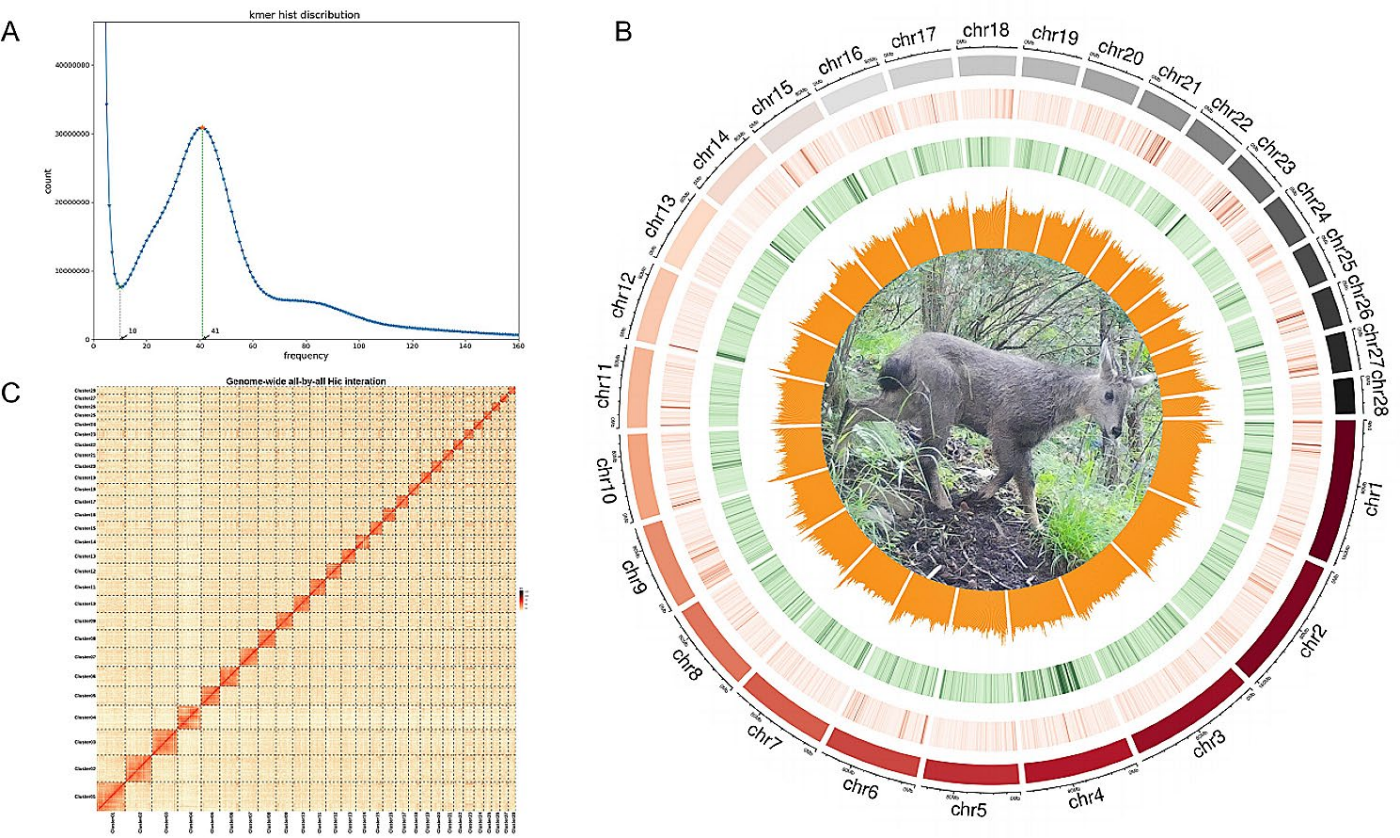
Genome analysis of *Naemorhedus goral*. **A**: Genome survey using 17-mer analysis. **B**: Circos plotting from outer to inner: chromosome (Mb), DNA, LINE and simple repeat content, GC content, gene density, goral photoed by camera traps in Makehe, Qinghai province, China. C: Hi-C interaction heatmap

Using PacBio sequel II long-read sequencing, we obtained 118.07 Gb (~ 46X) of original data (Table 1), with subreads of an average length of 11,357 bp and an N50 length of 16,369 bp. The NGS paired-ended sequenced raw reads were 167.75 Gb (~ 65X) (Table 1; Table S1). The assembled goral genome size was estimated as 2.40–2.63 Gb, (k = 17, Table S2; Fig. 1A). The final polished genome assembly was ~ 2.59 Gb with a contig N50 of ~ 3.70 Mb, a scaffold N50 of 106.66 Mb and ~ 41.89% GC content (Table S3; Fig. 1B). 96.9% of 3,979 complete BUSCOs were identified, with a single-copy accounting for 95.3% (Table S4). After Hic-assisted attachment, 1423 scaffolds were successfully clustered and attached to 28 pseudo-chromosomes (Fig. 1B), with a Hi-C attachment rate of 96.67% (Table S5).

**Table 1:** Statistics of genome assembly and annotation of the chromosome-scale genome

Item	Category	Number
Sequencing data	PacBio CLR (Gb)	118.07
	Illumina short WGS (Gb)	167.75
	Hi-C (Gb)	157.56
	MGI RNA (Gb)	16.52
Assembly	Estimated genome size (Gb)	2.40–2.63
	Assembled genome size (Gb)	2.59
	Contig number	1472
	Contig N50 (Mb)	3.70
	Scaffold number	77
	Scaffold N50 (Mb)	106.66
	Longest scaffold (Mb)	179.09
Annotation	GC content (%)	41.89
	Repeat sequences (%)	49.99
	Number of protein-coding genes	20,145
	Number of functional annotated genes	20,130
	Average gene length (bp)	46096.02
	Average exon length (bp)	179.63
	Average intron length (bp)	5454.91
	Average exon per gene	9.15

A total of 3,900,817 repetitive homologous sequences with a size of ~ 1.29 Gb were identified, constituting 49.84% of the goral genome (Table S6). The transposable elements (TEs) occupied 48.37% of the genome, of which 41.92% were retroelements, consisting of long interspersed elements (LINEs, 37.07%), short interspersed elements (SINEs, 11.20%), and long terminal repeats (LTRs, 5.12%) (Table S6). Integrating the results of homology annotation, ab initio prediction, and transcriptome annotation, a final set of 20,145 genes (average gene length of 46,096.02 bp) was predicted in the genome, with an average of 9.15 exons per gene and average coding sequence (CDS) length of 1,643.75 bp (Table S7). Among these, 20,118 genes (99.87%) were loaded on chromosomes, with gene density (4.11–11.99 genes/Mb) and GC content (39.93–46.22%) positively associated with correlation value of 0.85 (*p*-value < 0.001) (Table S5; Fig. 1C). A total of 20,130 genes (99.93%) were functionally annotated based on at least one database, indicating a reliable annotation of the *N. goral* genome (Table S8).

### Phylogenetic analysis at the mitochondrial and genome level

**Fig. 2:**
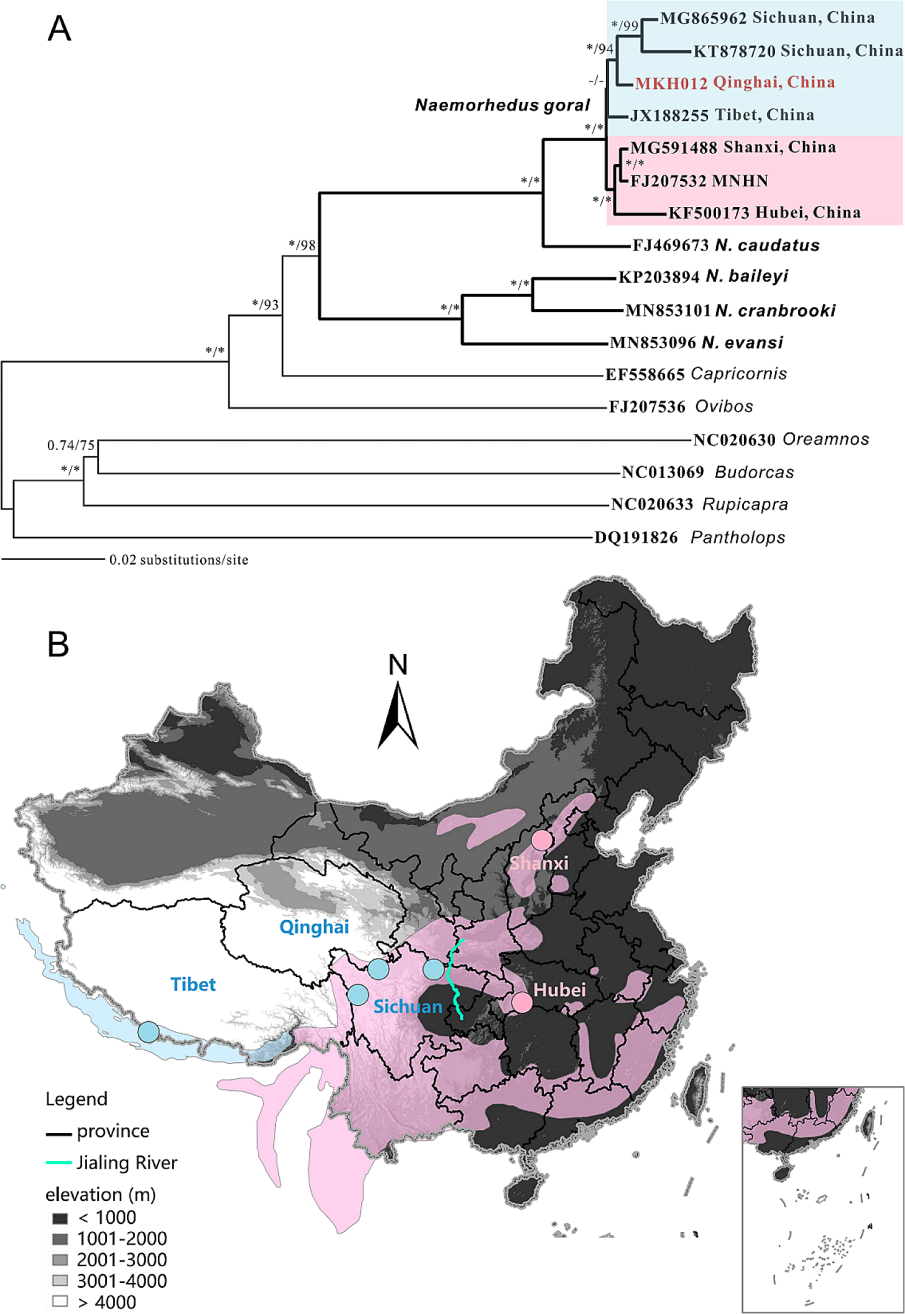
**A**: Phylogenetics at the mitochondrial genome level. *Naemorhedus* species are shown in bold. In other genera, one representative species was used for reconstruction. **B**: Distributions of *N. goral* (blue) and *N. griseus* (pink) assessed in IUCN, with collection localities corresponding to two clades colored in A

The size of the assembled mitochondrial genome of goral sample MKH012 was about 16,554 bp, sharing the sequence similarity from 98.54% with *N. goral* (GenBank accession No. KT878720) to 99.19% with *N. griseus* (GenBank accession No. JX188255) in NCBI database (Table S9). It was identified as Himalayan goral *N. goral* on the mitochondrial genome tree, a sister species of Long-tailed goral *N. caudatus* (Fig. 2A). The so-called Chinese goral *N. griseus* (KF500173 and FJ207532) was clustered with *N. goral* from Shanxi, instead of being monophyletic (Table S9). Two clades within *N. goral* were identified: the West clade in the southeastern margin of QTP (Tibet, Qinghai and Sichuan) and the East clade in the eastern China (Shanxi and Hubei) (Fig. 2B). This might indicate two evolutionary significant units under *N. goral*. Among the Caprinae species, goral clustered with serow and muskox, far from chamois and mountain goat which clustered with takin in another clade on the mitochondrial genome tree (Fig. 2A).

**Fig. 3:**
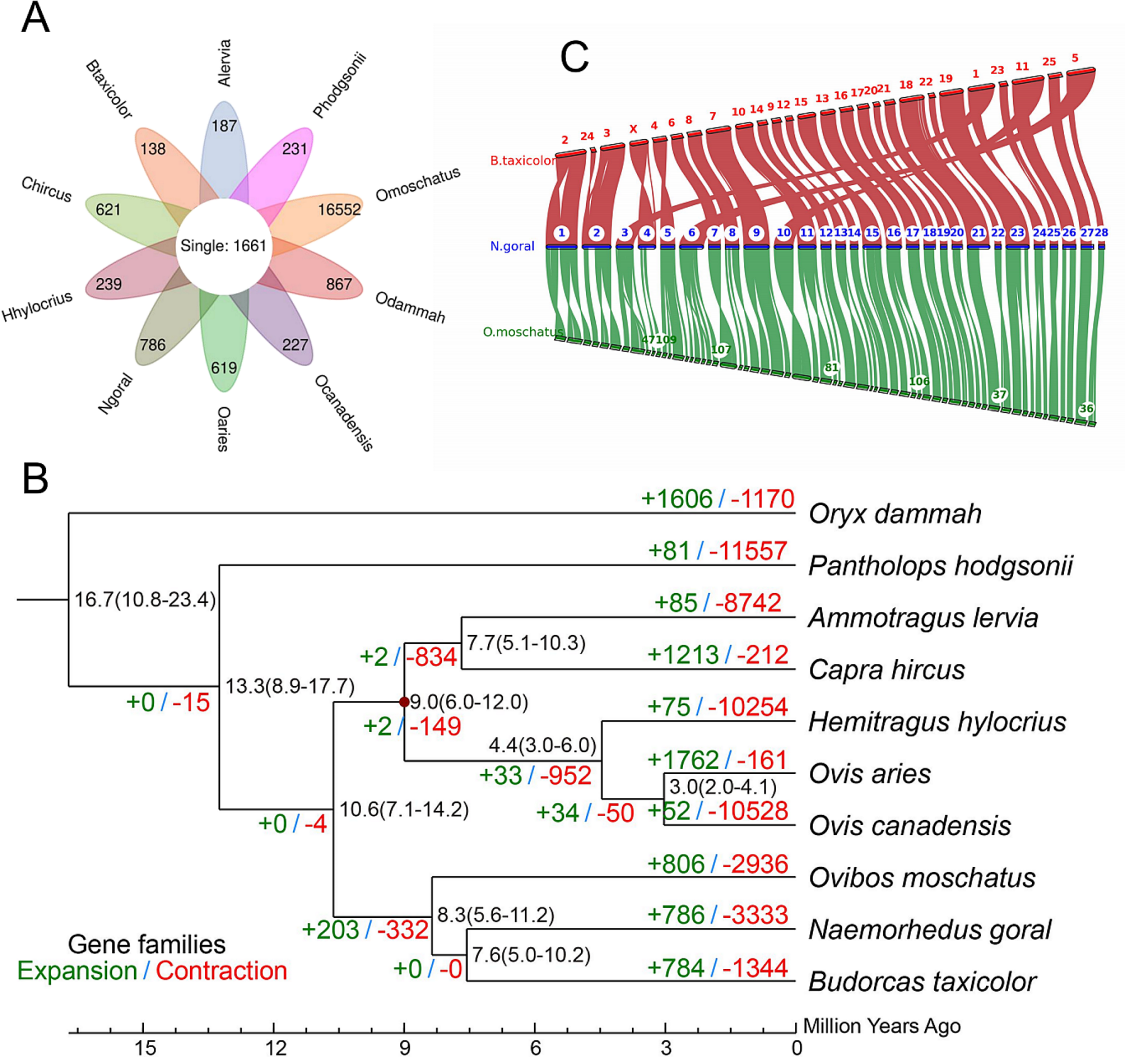
Orthologous genes and phylogenetic relationship among genomes of *N. goral* and other nine Bovidae species. **A**: Venn plot of single-copy genes and specific genes of each species. **B**: Phylogeny with expanded/contracted gene families and divergence time estimation. C: Collinear relationship of *N. goral*, *B. taxicolor* and *O. moschatus*

Gene families in goral were clustered with the other 9 Bovidae species on the genome tree (Table S10). Based on the identified orthologous gene sets, 1,661 single-copy orthologous genes were obtained and used for molecular phylogenetic analysis (Fig. 3A). On the reconstructed genome tree, goral clustered with muskox and takin, and was closely related to the latter (Fig. 3B), which was inconsistent with their phylogenetic relationship at the mitochondrial genome level. Molecular dating showed that the ancestor of goral and muskox had split from that of sheep and goat at ~ 10.6 Mya (95% HPD = 7.1–14.2), while the muskox showed a split at ~ 8.3 (95% HPD = 5.6–11.2) Mya from the proto-takin-goral stock. The divergence time between takin and goral was ~ 7.6 (95% HPD = 5.0-10.2) Mya.

### Gene family

**Fig. 4:**
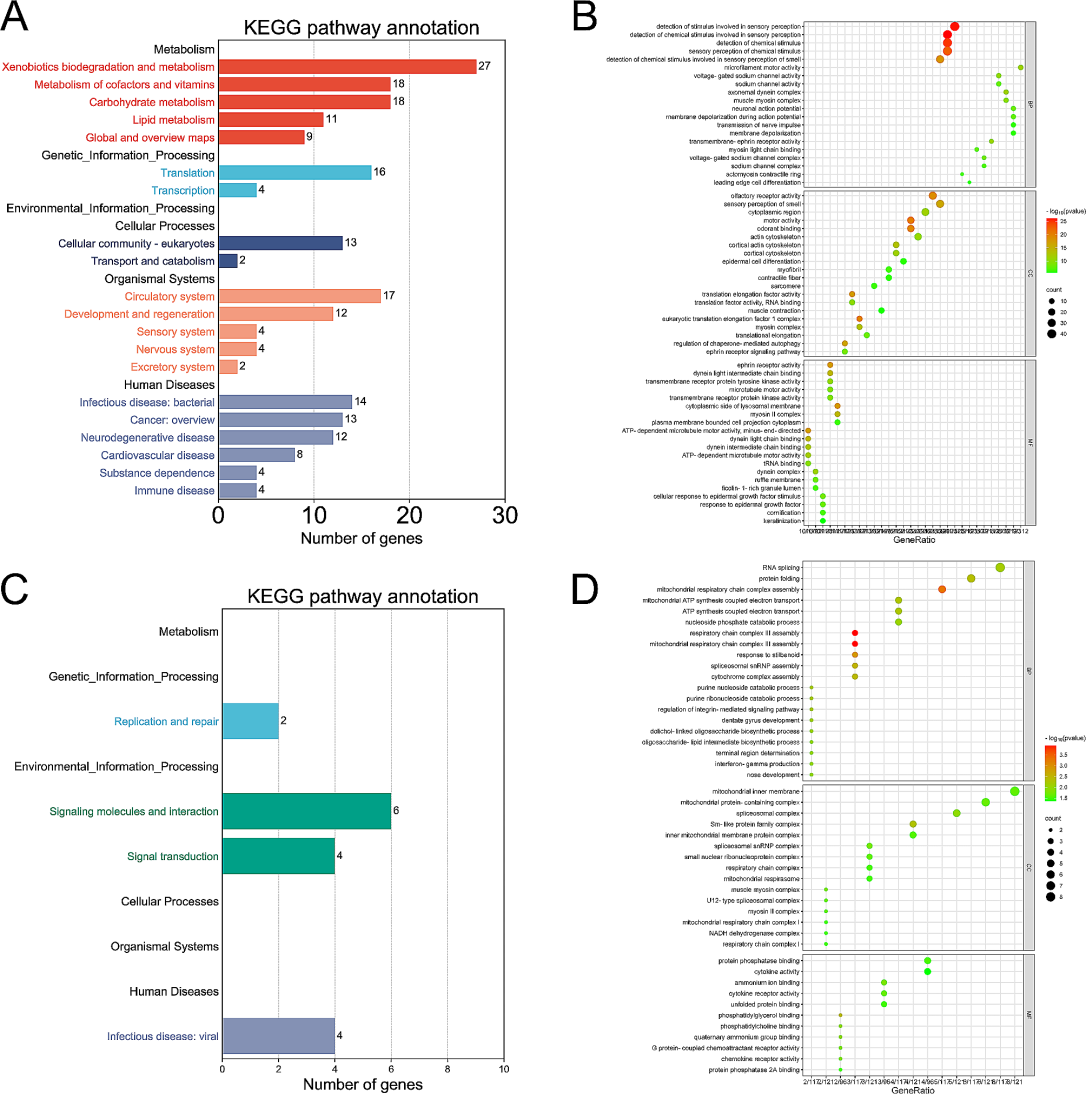
Enrichment analysis for expanded gene families by KEGG **(A)** and GO **(B)**, and for positively selected genes by KEGG **(C)** and GO **(D)**. The first 20 in each class were displayed. Value around each bar indicates number involved in each pathway

Compared the identified gene families among 10 Bovidae species on the phylogenetic tree, a total of 786 expanded and 3333 contracted gene families were revealed in the *N. goral* genome (Fig. 3B). After KEGG and GO enrichment analysis of the expanded orthologous groups, 17 significantly over-represented pathways and 280 significantly enriched GO terms were obtained with an adjusted *p*-value < 0.05 (Tables S11 and S12). Conducted by the branch-site likelihood ratio test with a *p*-value < 0.05, 144 positively selected genes (PSGs) in the *N. goral* lineage were identified (Table S13), 4 KEGG pathways and enriched GO terms were obtained with a significance of *p*-value < 0.05 (Tables S14 and S15). It should be noticed that the expanded gene families and PSGs with significant KEGG pathways are related to the metabolism of drugs and diseases, similarly to the significantly enriched GO terms (Fig. 4).

### Synteny analysis

A one-to-one corresponding relationship was observed between chromosomes of goral and its closest related takin and muskox (Table S16), with reverse complementary sequence in several chromosomes, i.e., cluster 4, also known as chromosome X and some chromosome fusions from muskox to goral and between goral and takin (Fig. 3C). Although at scaffold level, the muskox genome appeared to be nearly complete, correlated gene arrangements provide a valuable framework for inferring shared ancestry of genes and utilization of findings from one organism to another.

### Demographic history

**Fig. 5:**
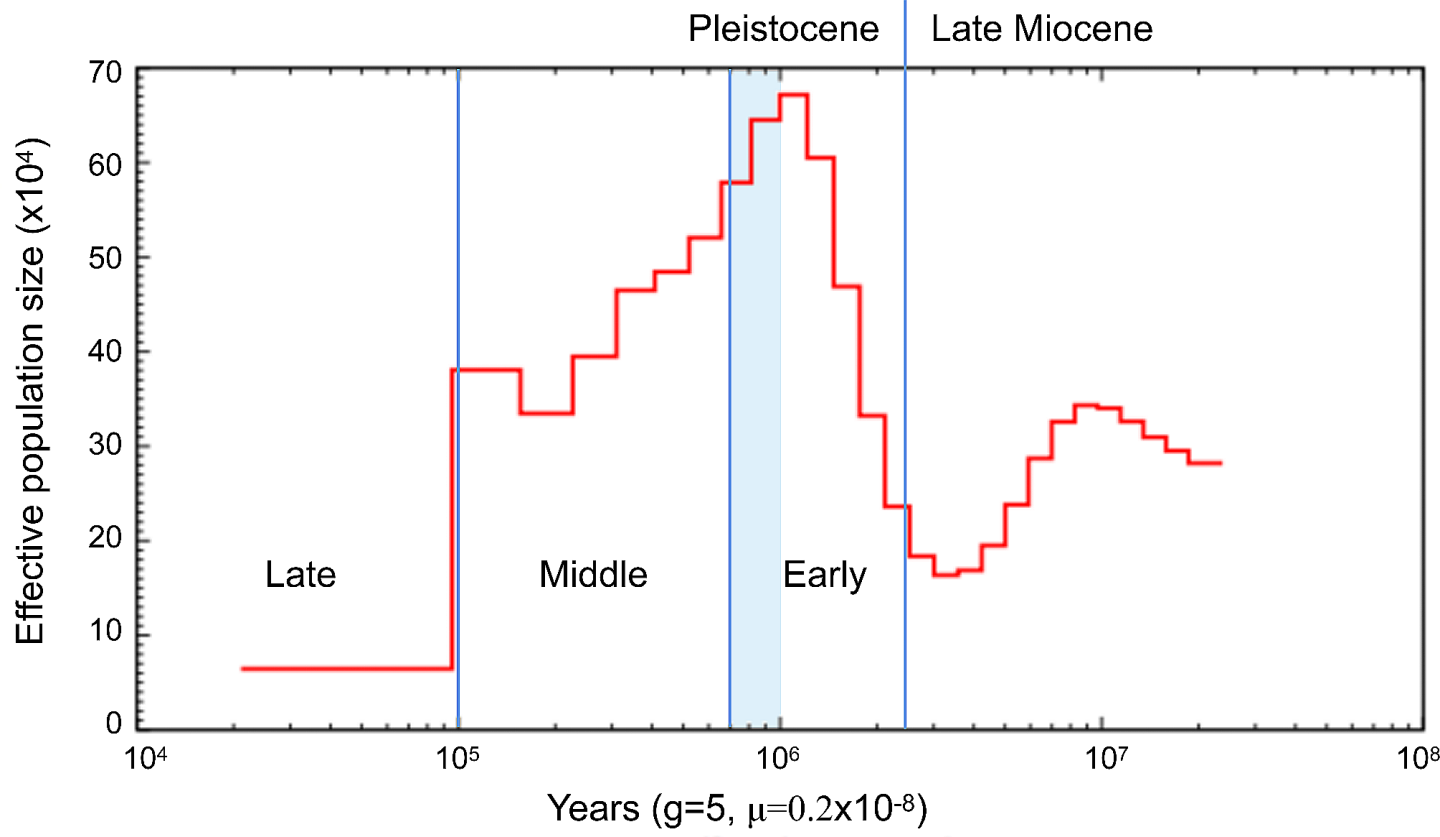
Demographic of goral. PSMC results showed the demographic history of goral, with a generation time (g) of 5 years and a mutation rate (µ) of 0.2 × 10 − 8 per site per generation. The time axis is logarithmically transformed

PSMC analysis suggested in effective population size (*Ne*) of goral an increase at the beginning of the Pleistocene, then a continuous decrease after ~ 1 Mya (the Xixiabangma Glaciation) and a significant drop by nearly 80-fold (Fig. 5).

## Discussion

We constructed the first high-quality chromosome-level genome of Himalayan goral and provided an important genetic resource for its evolution and conservation research. The genome size of *N. goral* is within the range of Caprinae species from 2.51 Gb (*O. americanus*) to 2.94 Gb (*C. hircus*) with the GC content similar to that of other Caprinae species ranging from 41.5% (*O. americanus*) to 43% (*Ovis canadensis*) (Table S3). Compared to most Caprinae genome assembled at chromosome-level, although the contig N50 of goral (~ 3.69 Mb) was smaller, the scaffold N50 reached ~ 106.66 Mb, larger than that of goat *Capra hircus* (~ 87.28 Mb), sheep *Ovis aries* (~ 101.27 Mb) and mouflon *Ovis orientalis* (~ 103.69 Mb), only smaller than that of takin (~ 109.75 Mb) (Table S3). Furthermore, the goral genome demonstrated an excellent collinearity connection with muskox and takin, indicating a high-quality assembly.

The assembled mitochondrial genome (16,554 bp) was highly similar to that in the GenBank database, showing a high credit. The mitochondrial genome phylogenetic result supported the *N. goral* (Himalayan goral) as an independent species, of which the *N. griseus* (Chinese goral) should be considered as a synonym, consistent with the previous suggestion [4, 18]. The two clades within *N. goral* might indicate two evolutionary significant units or subspecies. The distribution of the West clade expands from Tibet to Sichuan, and the East clade is restricted in eastern China. There is a possibility that the Jialing River is the distribution boundary (Fig. 2B), which probably distinct the subspecies of takin from Sichuan and Qinling [19].

The traditional tribes within the subfamily Caprinae, neither of the ‘goat-antelopes (Rupicarini)’ nor the ‘ovibovines (Ovibovini)’, are supported by the phylogenetic results in this study, due to the goral-serow-muskox clade far from takin-mountain goat-chamois clade on the mitochondrial genome tree and the goral nested into the takin-muskox clade on the genome tree. Groves and Shields have proposed to dissolve the Ovibovini as early as 1996, to which a similar conclusion has been yielded using mitochondrial DNA resources and widely discussed in the subsequent studies [10–14]. These two tribes are continuously used and even borrowed into the field of paleontology, such as the ‘late Miocene ovibovines’ [20] which is also highly debatable and not closely related to the extant ovibovine, phylogenetically [21]. They are so-called ‘rogue taxon’ with sensitive phylogenetic positions, thought to destabilize trees and present an obstacle to molecular phylogenetics [22]. The explanation deduced by Groves and Shields (1996) that “the rapid radiation of Caprinae has probably resulted in enough homoplasy to reduce the resolving power of this phylogenetic analysis” should be considered attentively [23].

On the genome level, the presence of the goral nests in the clade of takin and muskox provides a new insight into their evolution. It might be linked to the evolution of phenotype, gene family and speciation [24]. The split between the proto-goral-muskox-takin and proto-sheep-goat stock is around 10.6 Mya, and that between the muskox and the proto-takin-goral stock at ~ 8.3 is almost identical to the estimation from Li et al. (2022) [15]. The cladogenetic event among goral, takin and muskox occurred during the late Miocene (~ 11 − 5 Mya), when QTP experienced a third dramatic uplift, the entire plateau and all mountains surrounding the Tibet-Himalaya-Hengduan region had reached their current elevations, with consequent profound changes in environment and biodiversity through the influence of topography on atmospheric precipitation [25, 26]. Drying and cooling in central and northern Asia led to the transition of climates and ecosystems over large areas [27, 28], which might have caused the divergence of muskox restricted in a circarctic distribution, currently. As forest-dwelling species, takin and goral survived in the mountain-gorge landform in the south and east of the plateau, where the rapid speciation within genus was favored, closely related to the rapid uplift of the QTP and Hengduan Mountains in the uplift-driven diversification hypothesis. Correlated gene arrangements provide a valuable framework for inferring shared ancestry of genes and utilization findings from one organism to another. Nonetheless, at scaffold level, the muskox genome appeared to be nearly complete to analyze the corresponding relationship between chromosomes of goral and its closest related takin and muskox.

During the Pleistocene, the glaciation was active, especially in the middle (1.2–0.7 Mya) with a fundamental climate transition and environment change which have greatly influenced the evolutionary histories and distribution patterns of most extant species. Since the oldest Xixiabangma Glaciation (1.1–0.8 Mya), the *Ne* of goral has continued to decrease, although there was a brief stability (interglaciation, perhaps), it has ultimately dropped by nearly 80-fold. The similar history has been experienced by takin, milu and snob-nosed monkey [15]. However, only one biological replicate limits the power of PSMC analysis. It’s still not easy to understand why the significantly expanded gene families and PSGs are related to the metabolism of drugs and diseases. For goral, more resequencing replicates and depth analysis are needed to understand the evolutionary history.

## Materials and methods

### Sample collection and sequencing

The sample (MKH012) used in this study was a female goral, identified morphologically, which was naturally dead in March 2021 from Makehe National Nature Reserve, Banma County of Qinghai Province, China. The leg muscle was collected, transported in a car refrigerator (-20℃) and stored in an ultra-cold storage freezer at -80℃ in the College of Life Sciences, Qinghai Normal University.

High-quality DNA was extracted using the Genomic DNA extraction kit (Cat#13323, Qiagen, Germany). DNA’s Concentration was detected by Qubit Fluorometer. Sample integrity and purity were detected by Agarose Gel Electrophoresis. After quality assurance, the DNA was randomly fragmented by Covaris and selected by Agencourt AMPure XP-Medium kit to an average size of 200–400 bp and used for BGI-seq library preparation. The selected fragments were through end-repair, 3’ adenylated, adapters-ligation, and PCR Amplifying and the products were recovered by the AxyPrep Mag PCR clean up Kit. The double-stranded PCR products were heat-denatured and circularized by the splint oligo sequence. The single-strand circle DNA (ssCir DNA) was formatted as the final library and qualified by QC. Libraries were then subjected to the DNBSEQ-T7 platform for sequencing. To make sure reads reliable, NGS paired-ended sequenced Raw reads for genomic survey were first filtered using fastp (v.0.21.0; under default parameters) to remove low-quality reads, adapters, and reads containing poly-N [29]. PacBio libraries with insertions > 20 kb were prepared and sequenced using the Sequel II platform. The Hi-C library was prepared using the restriction endonuclease dpnII and subjected to the DNBSEQ-T7 platform for sequencing. All of the sequencing services were provided by the Haorui Genomics Technology Co., Ltd. (Xi’an, China).

### Genome size estimation and assembly

Genome size and heterozygosity were estimated by the *k*-mer analysis performed using NGS DNA data prior [30]. Briefly, cleaned reads were subjected to 17-mer frequency distribution analysis using the jellyfish v2.2.10 [31]. Then, the genome size was estimated with the 17-mer depth distribution from the 350-bp library cleaned sequencing reads in findGSE [32] and GenomeScope 2.0 [33], with the following equation: G = K-num/K-depth (where K-num is the total number of 17-mers, K-depth denotes the k-mer depth, and G represents the genome size). Further combining the simulation data results of Arabidopsis with different heterozygosity and the frequency peak distribution of 17 kmer, the heterozygosity and repeat content of goral genome were estimated.

Through comparative analysis, the unique read pair around the restriction endonuclease site was determined. By standardizing the restriction endonuclease sites on the genome sketch, Hi-C linkage is used as a measure of the degree of correlation between different Scaffolds. For a genome sketch with a karyotype of 2n, the Scaffold sequence of the sketch is clustered into n chromosomal groups using 3D-DNA (v201008) [34], and then visualized through the interaction signals of each chromosome, corrected through heatmaps. Finally, the Scaffold sequence with the determined sequence and direction is added to connect 100 N’s to obtain the final genome sequence at the chromosome level. The integrity of the assembly was evaluated by BUSCO (v5.0.0) with the dataset mammalia_odb9 [35]. To assess the continuity of the assemblies, the assembled genome was collinearly aligned with the high-quality genomes of goat (Saanen_v1) and sheep (ARS-UI_Ramb_v2.0) using Minimap2 (v2.17) [36].

### Repeats and gene annotation

The repetitive sequences in goral genome were identified using RepeatMasker (v1.323) [37] and RepeatProteinMask based on RepBase library (2018.10.26). A *de novo* repeat library was constructed using RepeatModeler (v2.0.1) [38], and then using RepeatMasker for annotating. All tandem repeat elements were recognized by Tandem Repeats Finder v4.07b (TRF). For gene annotation, based on the protein sequences of *Bos taurus* (GCF_002263795.2), *Capra hircus* (GCF_001704415.2), *Ovis aries* (GCF_016772045.1), *Cervus elaphus* (GCF_910594005.1), and *Mus musculus* (GCF_000001635.27) downloaded from NCBI, the protein-coding genes of goral were aligned using blast and Genewise (v2.2.1) [39] for homology annotation. AUGUSTUS (v3.3) [40] was used for ab initio prediction. The above prediction results were integrated by EVidenceModeler (v1.1.1) [41] into a non-redundant gene set. For the gene functional annotation, the protein-coding genes were aligned against several known public databases, including Swiss-Prot [42], TrEMBL (TRanslation of EMBL) [43], gene ontology (GO) [44] and Kyoto Encyclopedia of Genes and Genomes (KEGG) [45].

### Identification of homologous and orthologous gene set

To identify homologous relationships among goral and 9 assessed Bovidae species, including *Budorcas taxicolor* (GCF_023091745.2), *Ovibos moschatus* (GCF_021462335.1), *Ammotragus lervia* (GCF_002201775.1), *Capra hirus* (GCF_001704415.2), *Hemitragus hylocrius* (GCF_004026825.1), *Ovis aries* (GCF_016772045.1), *Ovis canadensis* (GCF_004026945.1), *Pantholops hodgsonii* (GCF_000400835.1) and *Oryx dammah* (GCF_014754425.2) (detailed in Table S10), their protein sequences were downloaded and aligned using OrthoMCL (v2.0.9) [46]. Proteins with homologs in the other 10 genomes were extracted as species-specific genes, including goral-specific exclusive genes and unclustered genes. Functional annotations of species-specific genes were performed using the GO and KEGG database.

### Phylogenetic analysis

We extracted the mitogenomic sequence of goral MKH012 and compared it to the assembled mitochondrial genome of Himalayan goral *N. goral* (MG591488, 16,559 bp in size) using LASTZ (v1.04.15) [47]. The optimal genomic sequence Contig34 (30,319 bp) was found and compared by Blat (v37 × 1) [48]. The repetitive boundary structure (1–13,765 and 16,555 − 30,319) indicated the goral’s complete mitochondrial sequence (1–16,554 bp segment of Contig34) was finally extracted. To construct phylogenetic tree, 10 mitochondrial genomes of *Naemorhedus* species as ingroups, 6 from *Capricornis*, *Rupicapra*, *Oreamnos*, *Ovibos*, *Budorcas*, and *Pantholops* as outgroups (Table S9) were downloaded, and aligned by MUSCLE (v3.8.31) [49] to exclude the ambiguous regions. Phylogeny was reconstructed using the maximum likelihood approach in PhyML 3.0 [50] and analyzed by Bayesian inference in MRBAYES 3.2 [51].

The coding sequences of the single-copy families were multiple-aligned using MUSCLE. The GTRGAMMA substitution model of RAxML (v8.2.10) [52] was used for the phylogenetic tree construction with 1000 bootstrap replicates. Based on the phylogenetic tree, MCMCtree (v4.8) [53] was utilized to compute the mean substitution rates along each branch and estimate the species’ divergent time. Three fossil calibration times were obtained from the TimeTree database [54], including divergence times of *Capra hirus* and *Ovis aries* (6-11.7 MYA). MP-EST [55] was used to construct root-cause and species phylogenetic trees. The branch length of 7.0 indicated that all gene trees support the triple structure of species phylogenetic tree with Pseudo-likelihood scores.

### Gene family analysis

The expansion and contraction of orthologous gene families were detected by CAFÉ v4.2.1 (-p 0.05 -t 10 -r 10,000) with a birth and death process to model gene gain and loss over a phylogeny [56]. Using R package clusterProfiler [57], both GO and KEGG functional enrichment analyses of the expanded gene families were performed. To identify positively selected genes in the goral lineage, we calculated average Ka/Ks values and conducted the branch-site likelihood ratio test using CodeML implemented in the PAML package Version 4.8 [58]. Genes with a *p*-value < 0.05 under the branch-site model were considered positively selected genes. Heatmap was plotted by online platform https://www.bioinformatics.com.cn [59].

### Synteny analysis

Correlated gene arrangements among *N. goral*, *B. taxicolor*, and *O. moschatus* provide a valuable framework for inference of shared ancestry of genes and for the utilization of findings from organism to organism. LAST software (v942) [60] was used to search species-pairwise synteny blocks, and JCVI (v1.2.7) utility libraries in MCscan (Python version) [61] were used to analyze and visualize the synteny between different genomes.

### Demographic history

At last, the historical effective population size (*Ne*) for *N. goral* was modeled using PSMC [62], with the default parameters of 64 atomic time intervals (-p “4 + 25 × 2 + 4 + 6”), a generation time of 5 years and mutation rate µ = 0.22 × 10^− 8^ mutations/site/year [63].

### Electronic supplementary material

Below is the link to the electronic supplementary material.

Supplementary Material 1

Supplementary Material 2

## Data Availability

The data generated in this study that support the findings, including the PacBio long reads, Illumina short reads, and genome assembly, have been deposited into the China National GeneBank DataBase (CNGBdb) with the project accession number CNP0003839. The genome assembly and annotation files are available under assembly accession number CNA0051961.
