# Supplementary material for: Chromosome-level genome provides insight into the evolution and conservation of the threatened goral (Naemorhedus goral)
Source: BMC Genomics. 2024 Jan 22;25:92. doi: 10.1186/s12864-024-09987-5 (PMC10804785; doi:10.1186/s12864-024-09987-5)
Supplement: Supplementary file 1 — Supplementary Material 1 [file 12864_2024_9987_MOESM1_ESM.doc]

**
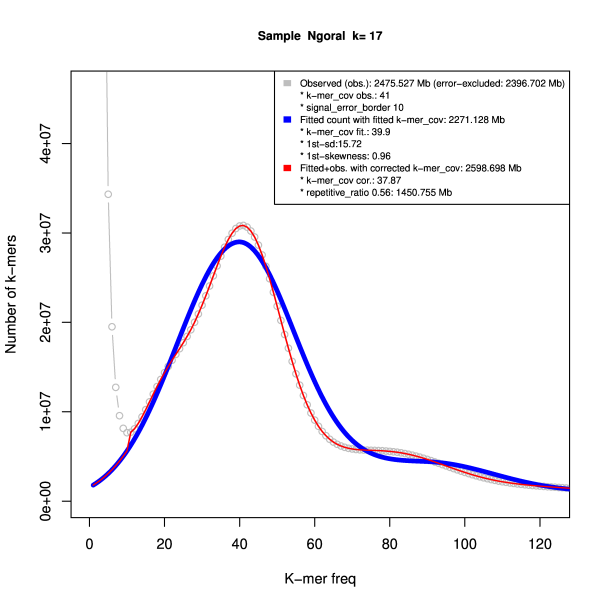

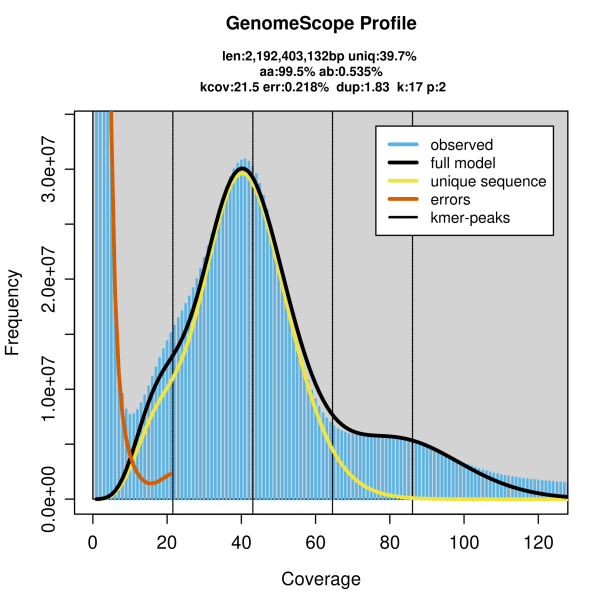
**

**Supplementary Figure S1.** Genome survey of *Naemorhedus goral* using 17-mer analysis by findGSE (left) and GenomeScope (right).

**
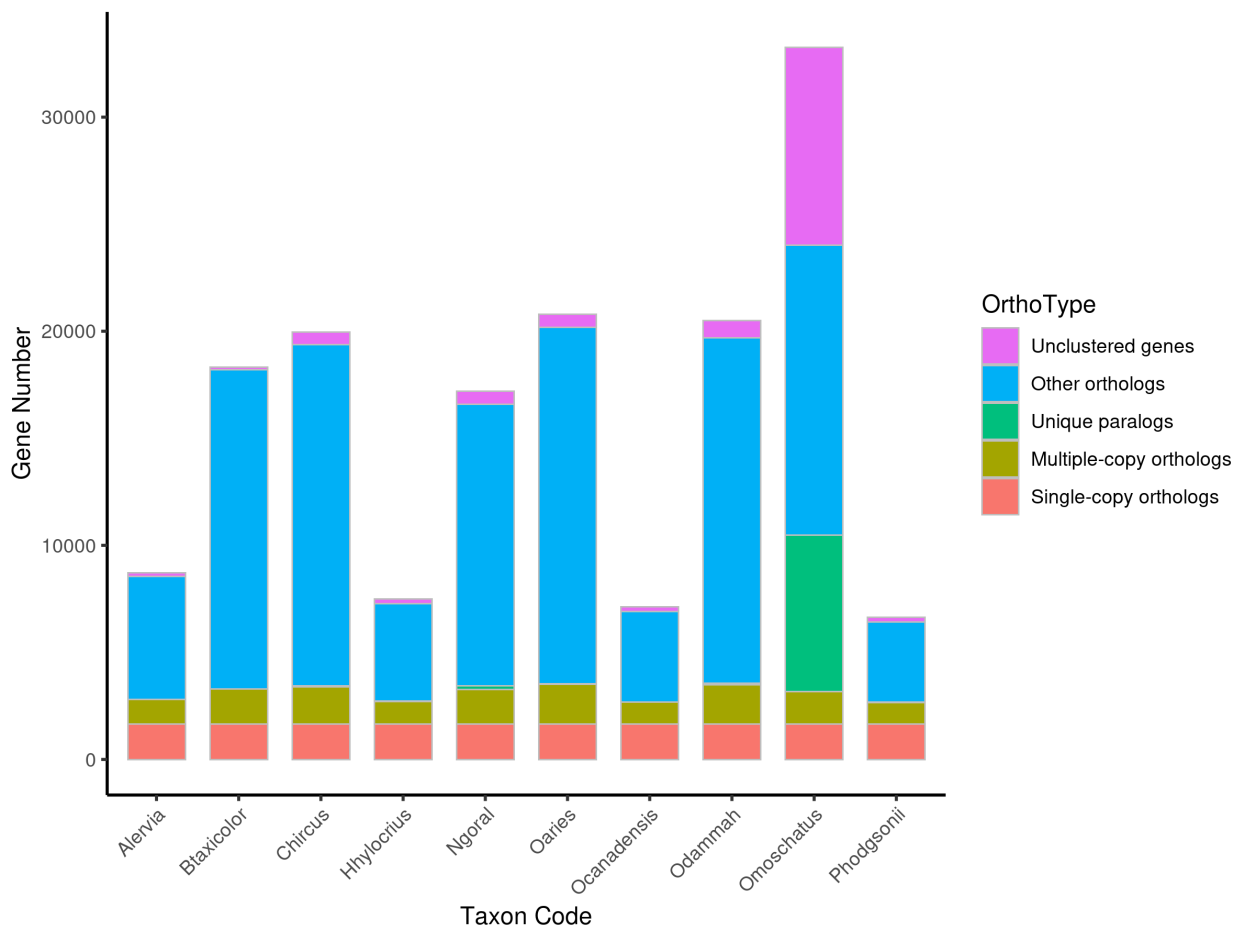
**

**Supplementary Figure S2.** Classification of orthlog types of each species.

**
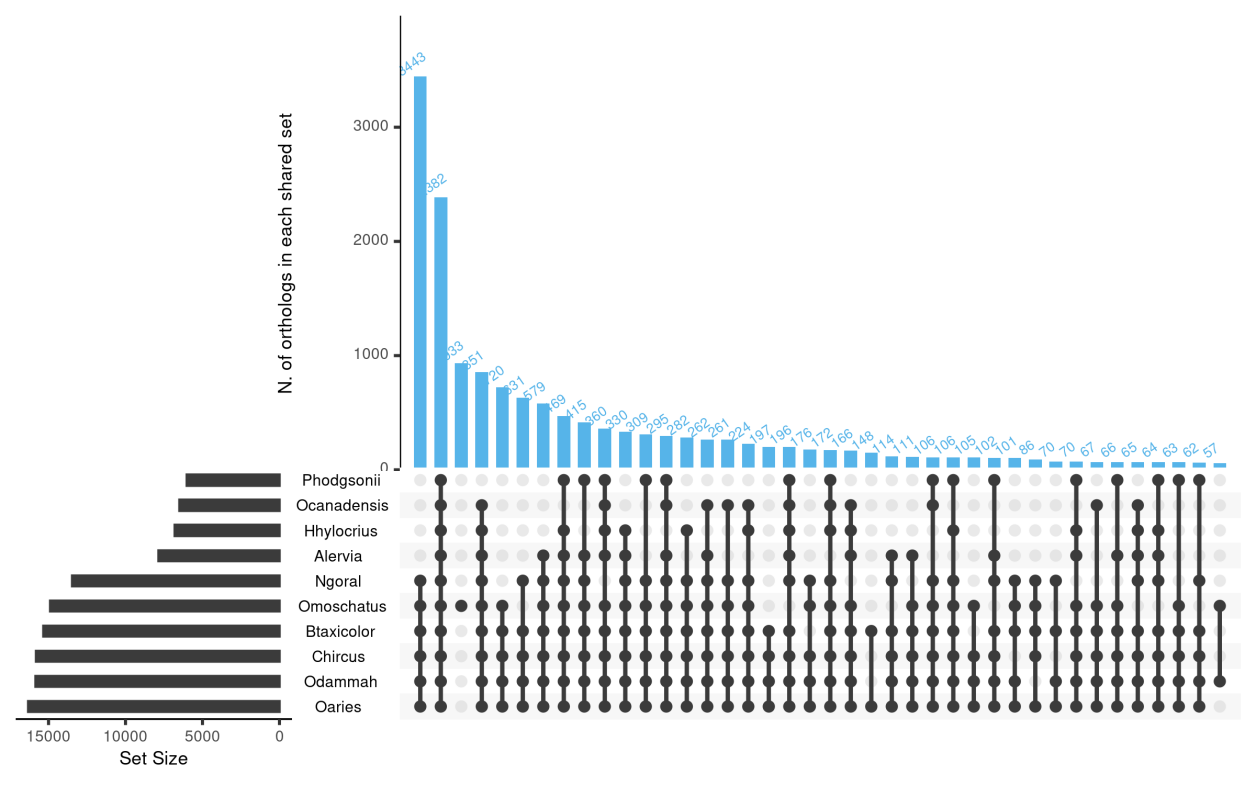
**

**Supplementary Figure S3.** Venn plot of homologous genes among species.

**
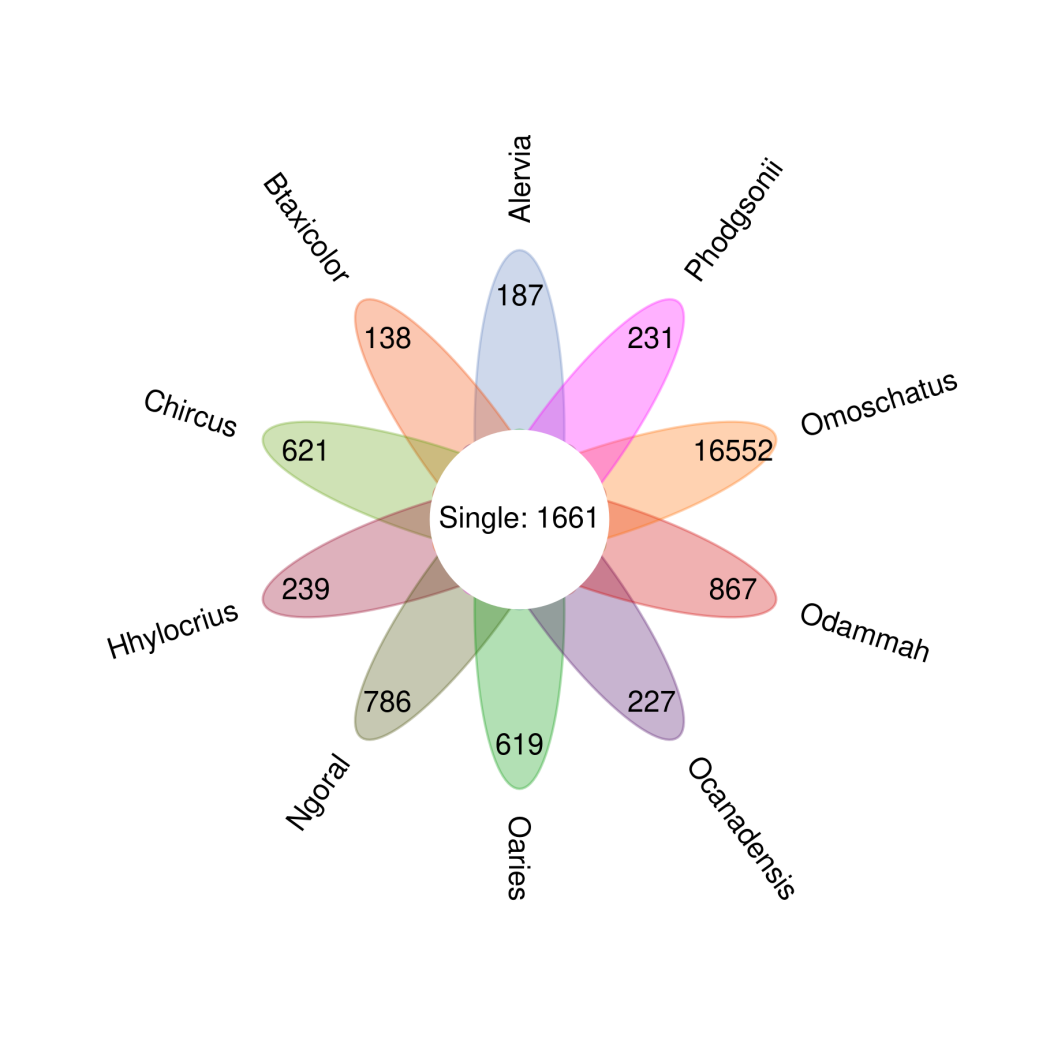
**

**Supplementary Figure S4.** Venn plot of single copy genes and specific genes of

each species.

**
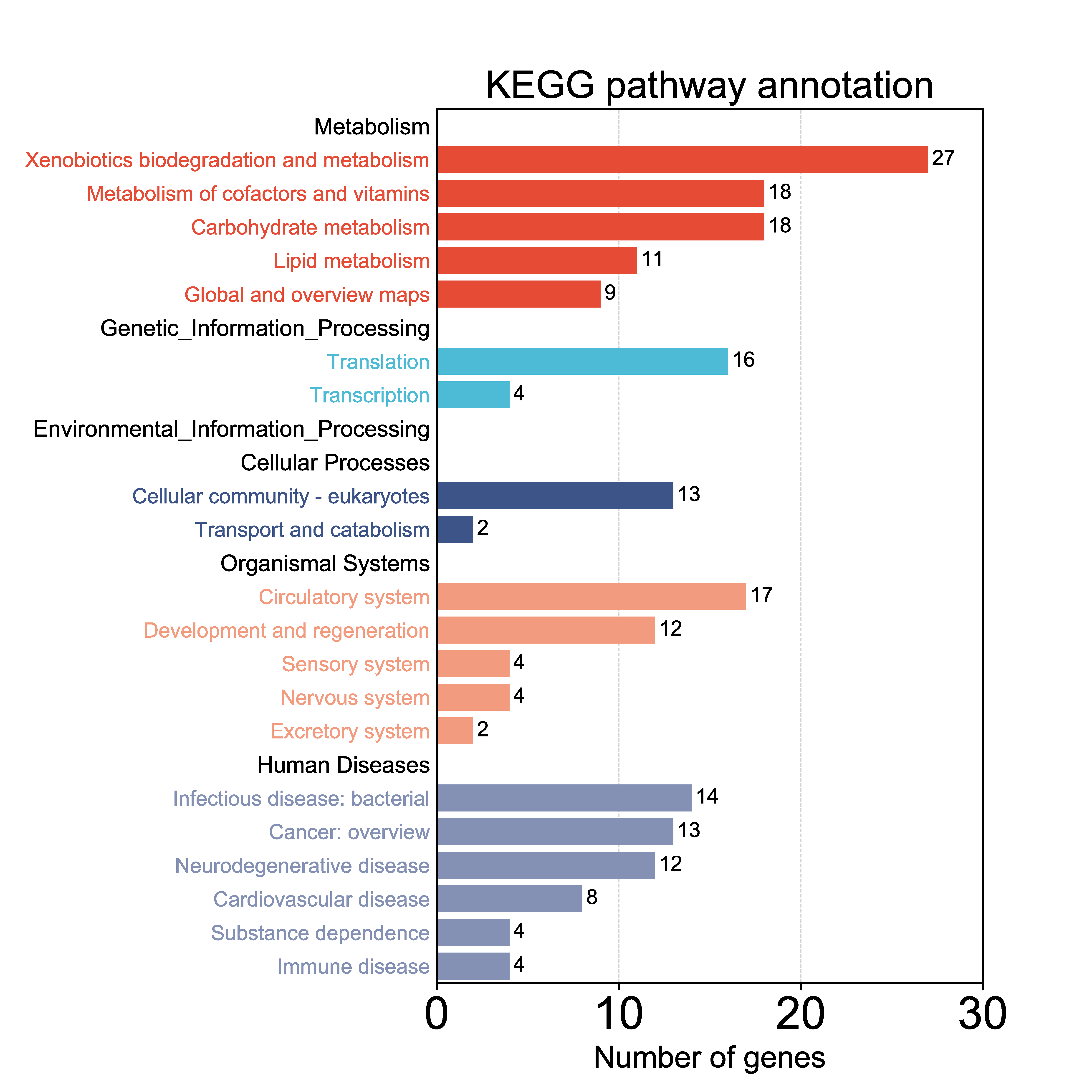

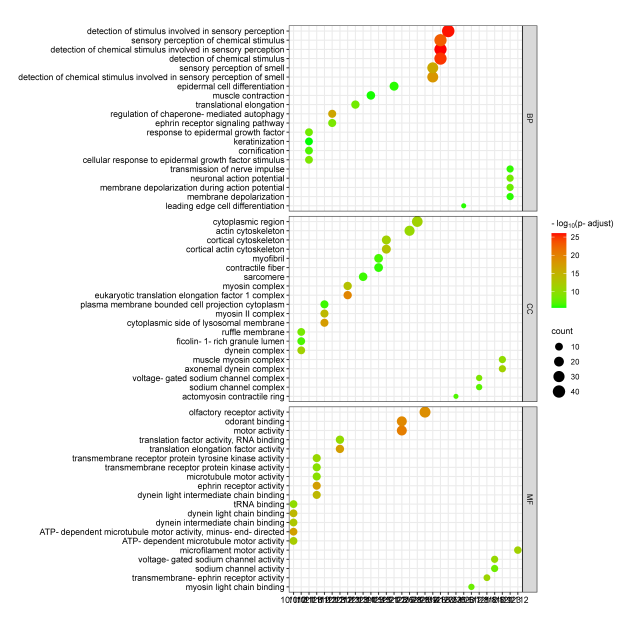
**

**Supplementary Figure S5.** KEGG and GO (the first 20 in each class were displayed) enrichment analysis of expanded gene families. Value around each bar indicates number involved in each pathway.

**
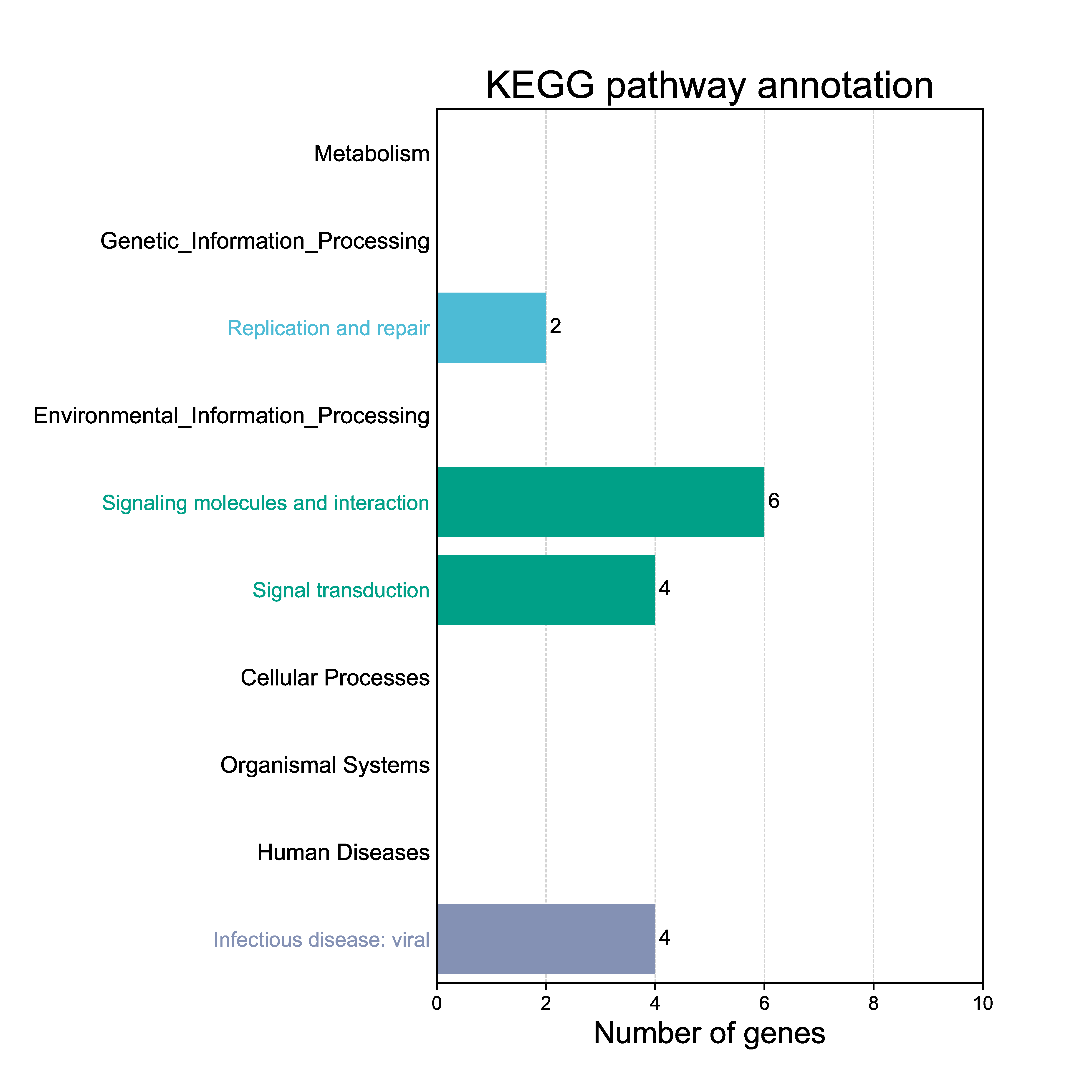

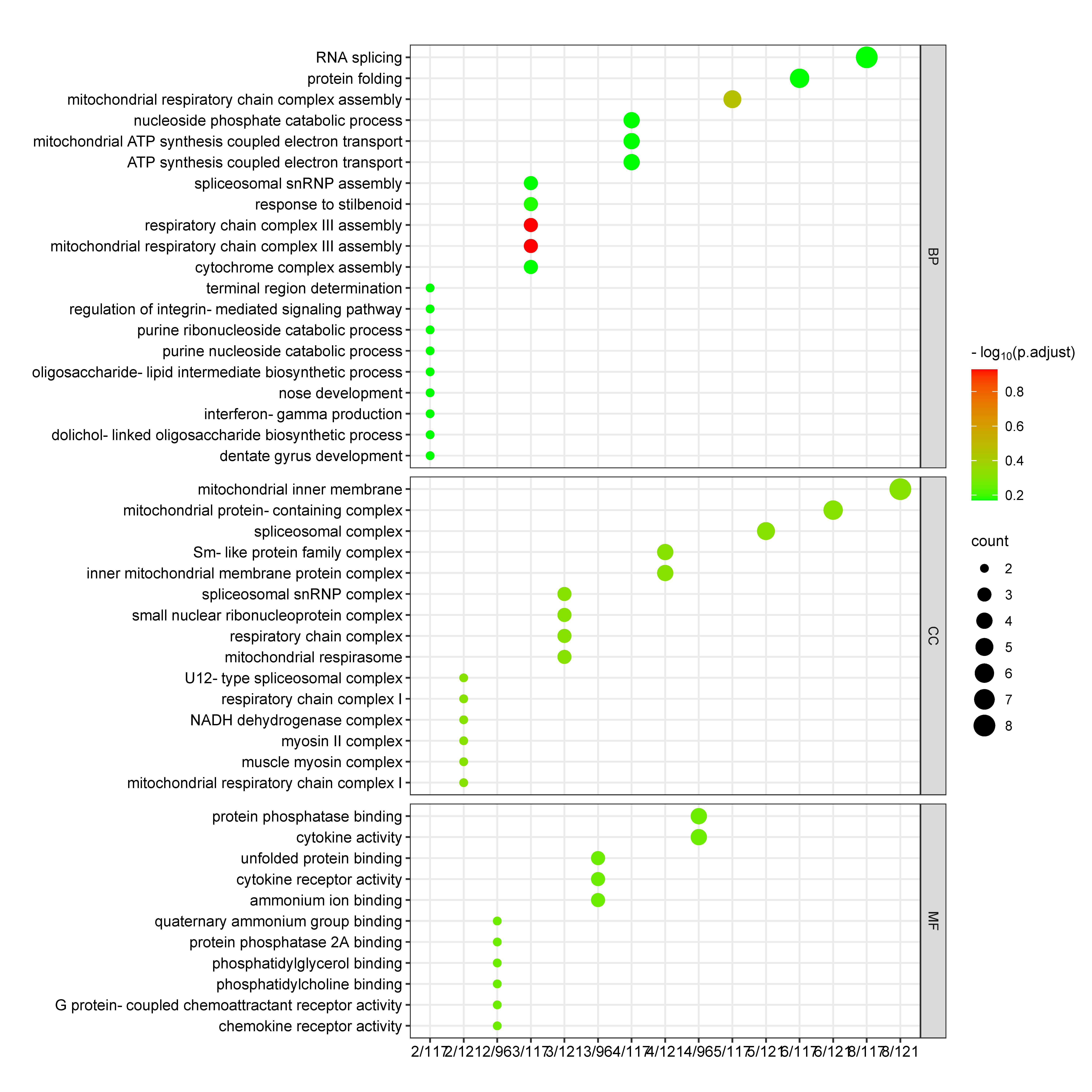
**

**Supplementary Figure S6.** KEGG and GO (the first 20 in each class were displayed) enrichment analysis of positively selected genes. Value around each bar indicates number involved in each pathway.
